# A randomized controlled trial to assess the central hemodynamic response to exercise in patients with transient ischaemic attack and minor stroke

**DOI:** 10.1038/jhh.2016.72

**Published:** 2016-09-29

**Authors:** J Faulkner, Y-C Tzeng, D Lambrick, B Woolley, P D Allan, T O'Donnell, J Lanford, L Wong, L Stoner

**Affiliations:** 1 Department of Sport and Exercise, University of Winchester, Winchester, UK; 2 Centre for Translational Physiology, University of Otago, Wellington, New Zealand; 3 Department of Surgery and Anaesthesia, University of Otago, Wellington, New Zealand; 4 Faculty of Health Sciences, University of Southampton, Southampton, UK; 5 School of Sport and Exercise, Massey University, Massey, New Zealand; 6 Neurology, Wellington Hospital, Wellington, New Zealand; 7 Department of Exercise and Sport Science, University of North Carolina at Chapel Hill, Chapel Hill, NC, USA

## Abstract

Early exercise engagement elicits meaningful changes in peripheral blood pressure in patients diagnosed with transient ischaemic attack (TIA) or minor stroke. However, central hemodynamic markers may provide clinicians with important diagnostic and prognostic information beyond that provided by peripheral blood pressure readings. The purpose of this single-centre, randomized, parallel-group clinical trial was to determine the effect of a 12-week aerobic exercise intervention on central and peripheral hemodynamic variables in patients with TIA or minor stroke. In this study, 47 participants (66±10 years) completed a baseline assessment, which involved the measurement of central and peripheral hemodynamic parameters, undertaken in the morning, in a fasted state. Participants were randomized to either a 12-week exercise or control group on completion of the baseline assessment. An identical follow-up assessment was completed post intervention. Central hemodynamic variables were assessed using an oscillometric device at both assessments. Analysis of covariance demonstrated a significant interaction for central and peripheral blood pressure and augmentation index (all *P*<0.05; *η*_p_^2^.09–.11), with the exercise group presenting lower values than the control group post intervention (118±17 vs 132±28 mm Hg for central blood pressure; 125±19 vs 138±28 mm Hg for peripheral blood pressure; 104±49 vs 115±67% for augmentation index). The present study demonstrates that participation in an exercise program soon after stroke/TIA diagnosis may elicit significant beneficial changes to a patient's central systolic blood pressure and augmentation index. This may positively impact upon the treatment strategies implemented by clinicians in the care of patients with TIA and minor stroke.

## Introduction

Blood pressure is an important risk factor for stroke,^1^ and is widely cited as a marker that needs to be controlled post stroke by pharmacological and lifestyle management.^2^ A recent meta-analysis with stroke and transient ischaemic attack (TIA) patients has shown that significant reductions in peripheral blood pressure responses may be evident following participation in behavioural interventions (that is, exercise, lifestyle management programs).^3^ However, the measurement of central hemodynamic parameters, including central systolic blood pressure (cSBP) and arterial wave reflection (that is, augmentation index, AIx), hold the potential to provide stroke clinicians with important diagnostic and prognostic information beyond that of peripheral blood pressure readings.

Peripheral blood pressures may not accurately reflect the effects of peak arterial blood pressure on centrally located organs such as the heart and brain.^4^ This is important because individuals who experience a stroke or TIA are at heightened risk of experiencing subsequent vascular events, whether from a cerebrovascular (that is, stroke/TIA) or cardiovascular (that is, myocardial infarction) origin.^5, 6^ Thus, the assessment of cSBP is particularly important as it reflects the stress and loading on the left ventricle and coronary arteries.^7, 8^ Yet to date, the effects of secondary prevention behavioural interventions on central blood pressure responses in stroke or TIA patients have not been assessed. As behavioural interventions which have incorporated exercise training, counseling and lifestyle education reduce the risk of subsequent cardiac events in patients with stroke,^3^ further research is necessary to elucidate the underlying central hemodynamic response to these types of intervention for patients with stroke or TIA.

The purpose of the current randomized control study was to determine the effect of implementing an exercise program soon after minor stroke/TIA diagnosis on cSBP and AIx. It was hypothesised that those individuals who engaged in the exercise program would demonstrate greater changes in central hemodynamic responses compared with those individuals randomized to a usual care control group.

## Methods

### Participants

**Figure 1:**
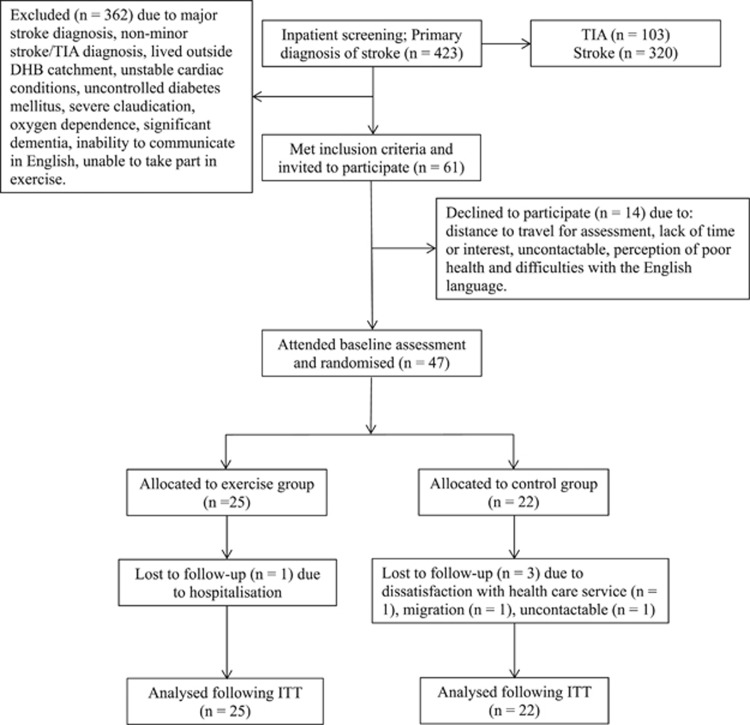
Participant recruitment.

Forty-seven participants (Male, *n*=35; 66±10 years; 90±19 kg; 173±7 cm; Female, *n*=12; 64±12 years; 80±20 kg; 159±4 cm) diagnosed with either TIA or minor stroke completed the baseline assessment, of which 43 took part part in a post-intervention assessment (Figure 1). Recruitment commenced in September 2013 and lasted 12 months with participants being randomized to either an exercise or usual care control group (exercise group, *n*=25; 66±12 years; 88±19 kg; 168±10 cm; control group, *n*=22; 68±10 years; 90±21 kg; 171±8 cm). Follow-up (post-intervention) assessments were completed between December 2013 and December 2014.

TIA and minor stroke diagnosis was based upon criteria from the New Zealand assessment and management guidelines.^9^ At the time of hospital presentation within the inpatient setting, stroke severity was assessed using the National Institutes of Health Stroke Scale, functional severity was assessed using the Barthel Index, and stroke risk of TIA patients was assessed using the ABCD^2^ score. Participants were eligible if they had been diagnosed with their first high-risk TIA (ABCD^2^ score of ⩾2) or minor stroke (defined as an National Institutes of Health Stroke Scale of ⩽5), lived within the local district health board catchment, and if they did not meet exclusion criteria (Figure 1). Exclusion criteria were: unstable cardiac conditions, uncontrolled diabetes mellitus, severe claudication, oxygen dependence, significant dementia, inability to communicate in English or unable to take part in exercise. All participants complied with drug treatment and standardised therapy in accordance with stroke physician recommendations. The trial was approved by New Zealand's Central Regional Health and Disabilities Ethics Committee and registered with the Australian and New Zealand Clinical Trials Registry (Trial Registration Number: ACTRN12613000869774), and conformed to the guidelines established in the *Declaration of Helsinki*. Written informed consent was obtained before participation.

### Sample size

On the basis of the findings of Woolley *et al.*,^10^ and when examining changes in peripheral systolic blood pressure (pSBP) in TIA patients following participation in an exercise intervention, and when using a two-sided 5% significance level and a power of 80%, a minimum sample size of 19 participants per group was calculated.

### Study design

The study was a single-centre, randomized, parallel-group design. TIA or minor stroke was confirmed by a specialist stroke physician at Wellington Hospital within 7 days of symptom onset. Eligible participants were invited to attend a baseline assessment conducted within a thermoneutral laboratory (21–22 °C) typically between 0700 and 1000 hours All participants were fasted (overnight fast), euhydrated, had abstained from caffeine and supplement intake during the morning, and from strenuous physical activity and alcohol consumption for 24 h before assessment. Following completion of a health history questionnaire, and 15 min of quiet supine rest, central blood pressure responses were obtained with the participant in the supine position. Identical assessments were completed post-intervention.

On completion of the baseline assessment, participants were randomized to either an experimental group (usual care plus participation in a 12-week aerobic exercise intervention) or to a control group (usual care, only) using computerized random numbers. Details pertaining to each allocated group were provided on a piece of paper contained within sequentially numbered, opaque sealed envelopes. The randomization procedures were prepared by an investigator with no clinical involvement in the trial. Although participants and the health and exercise practitioner were aware of the allocated treatment condition, data analysts were kept blinded to the allocation.

Subjects randomized to the exercise program completed twice weekly, group-based (three to five participants) exercise sessions for 12 weeks. Exercise was prescribed on a one-to-one basis by health and exercise practitioners. In accordance with recommendations for moderate physical activity participation, participants completed 30–60 min of aerobic exercise at each exercise session.^11^ This included up to 30 min of continuous walking and up to 30 mins of continuous cycling, with an interim period of ~5 min of passive recovery between bouts. Blood pressure, heart rate and ratings of perceived exertion were measured prior to, during and following each bout of aerobic exercise. Participants commenced their exercise program at 50% of age-predicted maximal heart rate during all aerobic exercise tasks. The exercise intensity and/or volume typically increased by ~5% each week, up to a maximal heart rate training intensity of 90% of age-predicted maximal heart rate. The rate of progression was dependent upon how the subject felt during each session. Subjects were instructed not to exercise beyond an ratings of perceived exertion of 15 (‘hard' feeling of exertion) during both walking and cycling exercise.^12^ Exercise practitioners ensured that subjects did not exercise above 90% of their age-predicted maximal heart rate.

The control group (usual care) received standard secondary prevention and educational information from the hospital on discharge and were requested to adhere to their prescribed medication for the duration of the study.

### Pulse wave analysis

An oscillometric device (BP^+^, Uscom Lts, Sydney, NSW, Australia) was used to conduct pulse wave analysis, from which central and peripheral blood pressures and AIx were derived. The AIx% is an independent predictor of cardiovascular risk and mortality.^13^ Pressure waveforms were recorded on the upper arm, following standard manufacturer guidelines.^14^ Briefly, the BP^+^ incorporates an oscillometric blood pressure module which complies with the Association for the Advancement of Medical Instrumentation (AAMI SP10) requirements and receives an A/A rating from the British Hypertension Society evaluation protocol.^15^ Each measurement cycle (~40 s) records brachial blood pressures and then one set of suprasystolic (~30 mm Hg>systolic) recordings for 10 s. The suprasystolic pressure signals were recorded by a high-fidelity pressure transducer, and the central pressure waveform was derived in the time-domain from the relationship between the total oscillatory pressure in the aorta and the total oscillatory pressure under the occlusion cuff.^15, 16^ This estimation was scaling-independent (that is, the estimated aortic wave shape did not depend on brachial blood pressure). AIx% was calculated using the formula: AIx%=(P3 – P0)/(P1 – P0), where P0 is pressure at the onset of the pulse (diastolic), P1 is peak pressure of the incident wave (systolic), P3 is peak pressure of the reflected wave. This index describes the relative height of the reflected pressure wave when compared with the incident waveform. Only recordings with a high signal quality were accepted. Signal quality was assessed using the signal to noise (S/N) ratio (in decibels), where S/N values >3 dB were considered acceptable. Two measurements were taken, with 5-min interval. If AIx varied by >4% or blood pressures by >5 mm Hg, a third recording was made and the two closest recordings were averaged.

### Statistical analysis

Independent samples *t*-tests (age, certain lifestyle factors) and Pearson Chi-squared tests (descent, family history of cardiovascular disease (CVD), personal history of CVD, signs and symptoms of CVD, medication) were used to compare baseline data between conditions (exercise, control). A similar analysis was used to assess central and peripheral hemodynamic properties (that is, cSBP, pSBP, peripheral diastolic blood pressure, central pulse pressure, peripheral pulse pressure, AIx) at baseline, and to assess medication use post-intervention. To assess the effect of the exercise intervention on the aforementioned central and peripheral hemodynamic properties, and following checks for normality to determine that these outcome variables were parametric, a series of a-priori repeated-measures analysis of covariance (Test [baseline, post intervention] by Condition [exercise, control], with baseline inserted as a covariate) were conducted. An intention-to-treat analysis was used on all repeated-measures statistical procedures, whereby the last recorded data from a participant's subsequent assessment was carried forward and used in place of any missing assessments thereafter. Partial eta squared (*η*_p_^2^) was used to demonstrate the strength of the effect of exercise on the various outcome measures with. 0099,. 0588 and. 1379 representing a small, medium and large effect, respectively.^17^ Partial eta squared was calculated using the following formula:


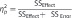


Whereby, SS_Effect_ is the estimated variance for a given outcome measure, and SS_Error_ is the error variance that is attributable to the effect. Alpha was set at 0.05. Statistical analyses were performed using Statistical Package for Social Sciences version 22 (SPSS, Inc., Chicago, IL, USA). All data are reported as means (s.d.), unless otherwise specified.

## Results

### Participant recruitment, adherence and recurrent TIA risk

There were no differences in subject characteristics, medication use or CVD risk factors at baseline between groups (*P*>0.05; Tables 1 and 2 and Supplementary Table). Baseline assessments were undertaken 8±3 days post diagnosis. Mean medication use post-intervention was approaching significance (*P*=0.09) with participants in the exercise group typically taking less medication compared to those in the control group (Mean difference (95% CI); 0.67 (−1.48 to 0.13); Table 2). Participants randomized to the exercise program attended, on average, 96% of the available sessions. There were no adverse events from participation in the exercise program.

### Central and peripheral hemodynamic variables

Table 3 summarises the mean values for the central and peripheral hemodynamic properties measured at baseline and post intervention for both exercise and control conditions. There were no statistical differences in any of the peripheral or central hemodynamic variables reported at baseline between the exercise and control group (all *P*>0.05). Analysis of covariance demonstrated a significant interaction for cSBP, pSBP and AIx (all *P*<0.05), with the exercise group presenting lower values than the control group post intervention. A significant interaction was also observed for peripheral pulse pressure and central pulse pressure (both *P*<0.05). An increase in pulse pressure was observed for the control group between baseline and post intervention, but a decrease was observed with the exercise group.

## Discussion

This is the first study to demonstrate that cSBP and AIx are significantly reduced in participants with minor stroke and high-risk TIA following participation in an exercise intervention, soon after diagnosis. In the present study, despite similarities in baseline demographics, the 7% improvement in cSBP following participation in a 12-week exercise program equated to a large effect size (*η*_p_^2^=0.11). The significance of this finding is further enhanced when considering that there was a large reduction in medication use for those participants randomized to the exercise group, and when considering that there were no changes in cSBP for those individuals who received usual care.

Of the various CVD risk factors that are often assessed in patients with minor stroke and TIA following engagement in an exercise program, peripheral blood pressure responses have received the greatest attention.^3, 18, 19, 20, 21^ However, it has been suggested that peripheral blood pressures may not accurately reflect the effects of peak arterial blood pressure on centrally located organs,^4^ nor the stress and loading on the left ventricle and coronary arteries.^7, 8^ Accordingly, in the present study, the significant reduction in cSBP is of pragmatic importance for this population group, as individuals diagnosed with stroke and TIA are at a heightened risk of experiencing further cardiovascular complications.^5, 6^ It is interesting to note that the 9 mm Hg mean reduction in cSBP is greater than that reported in a recent meta-analysis, whereby, on average, a 4 mm Hg reduction in peripheral blood pressure is suggested to occur following lifestyle and behavioural interventions.^3^ The prescription of exercise at a moderate- to high-intensity in the current study may be an underpinning reason for this difference. Nevertheless, as the reduction in pSBP was identical to that reported for cSBP for those in the exercise group, the ecological benefit of monitoring cSBP may be somewhat reduced. In the current study, the exercise group also demonstrated a significant reduction in central and peripheral pulse pressure in comparison to the control group (*η*_p_^2^=0.10 to 0.14). Because the statistically similar diastolic blood pressures reported between conditions (exercise and control) and assessment sessions (baseline, post-intervention), the ~5 mm Hg reduction in pulse pressure for the exercise group is largely derived by the aforementioned changes in systolic blood pressure (Table 3).

A further novelty of the present study relates to the change in AIx; an indicator of arterial wave reflection.^22^ In this study, participants who took part in the exercise program demonstrated a 15% reduction in AIx (Table 3). This finding is of significant importance for those with stroke/TIA as AIx is an indicator of systemic arterial stiffness^23^ and demonstrates the contribution made by the reflected pressure wave to the ascending aortic pressure waveform.^22^ Conversely, and unexpectedly, AIx increased for the control group. Increased arterial wave reflections have been reported to have adverse effects on ventricular afterload and coronary perfusion, have been significantly correlated to the degree of coronary artery disease,^24^ and independently predict cardiovascular risk and mortality.^25^ When considering that stroke and TIA patients have an elevated risk of experiencing cardiovascular and cerebrovascular events post stroke/TIA,^1^ the measurement of central hemodynamic parameters, including cSBP and AIx, could provide clinicians with important diagnostic and prognostic information beyond that provided by traditional peripheral blood pressure readings. From a pragmatic standpoint, oscillometric devices, like that used in the current study, can provide clinicians with a quick, non-invasive and valid assessment of such hemodynamic parameters.^25, 26, 27, 28^ Despite this study being sufficiently powered, it is plausible that the observed differences in AIx between the experimental and control groups may be associated with the relatively small sample size and thus, future research should assess such hemodynamic parameters in a larger TIA/minor stroke population.

A recent study reported that the monitoring of central blood pressure, compared with peripheral blood pressure, aided in the management of hypertension and led to decreased medication use without adverse effects of left ventricular mass.^29^ Therefore, future research should assess whether the monitoring of central hemodynamic variables in those with minor stroke or TIA may lead to similar, positive clinical outcome changes, which may lead to more targeted treatment strategies for these population groups. However, it is important to recognize that in the current study there are some important findings with regards to medication use (Table 2). At the time of follow-up, participants who engaged in the exercise program were shown to be taking significantly fewer anti-thrombotic medications, and there was a trend for taking fewer medications overall, compared to those randomized to the usual care control group. When considering that medicinal management is primarily carried out to have an impact on the cardiovascular system, this finding is pertinent, and reflects the value of engaging patients in an exercise program soon after diagnosis.

In conclusion, the present study has demonstrated that participation in an exercise program soon after stroke/TIA diagnosis may elicit significant changes in cSBP and AIx. It may be important for stroke clinicians to consider central hemodynamic parameters at diagnosis and during follow-up assessments as these parameters may provide important information beyond that provided by traditional peripheral blood pressure readings. As automated oscillometric devices can provide a valid, quick and non-invasive assessment of central hemodynamic parameters, clinicians could consider the use of such measurements in providing more targeted treatment strategies for those with TIA or minor stroke.


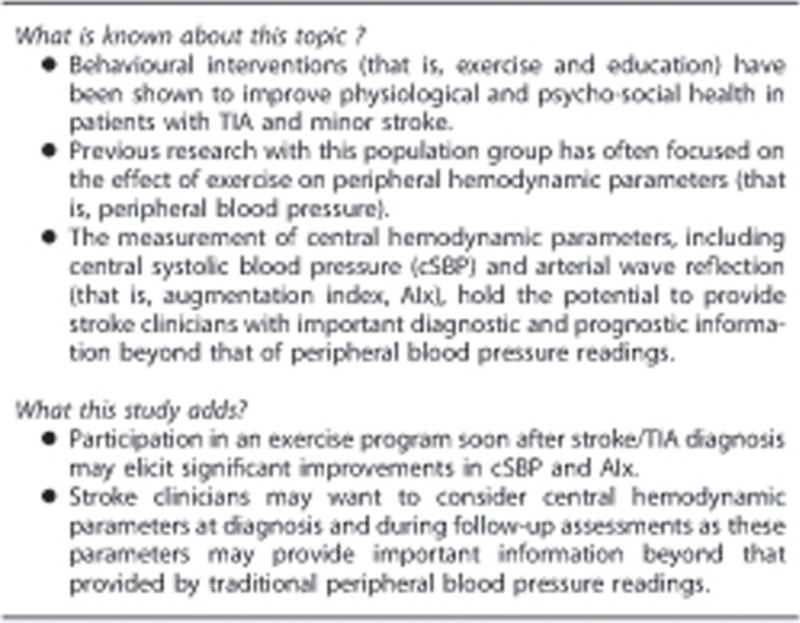


## Figures and Tables

**Table 1:** Baseline demographics for both exercise and control groups

	*Exercise*		*Control*		P*-value*
	*n*	*%*	*n*	*%*	
Participants (*n*)	25	53	22	47	
Age (y)	66±12		68±10		0.606
					
*Gender (n)*					
Male	17	68	18	82	0.288
Female	8	32	4	18	
					
*Stroke classification*					
Ischaemic stroke	8	32	8	36	0.759
TIA	17	68	14	64	0.759
					
*Family history of CVD*					
Myocardial infarction	10	40	10	45	0.713
Stroke	14	56	10	45	0.481
					
*Personal history of CVD*					
Hypertension	13	52	17	77	0.105
High cholesterol	12	48	15	68	0.220
Diabetes	4	16	8	36	0.134
Heart problems	9	36	8	36	0.938
Artery diseases	1	4	0	0	0.344
					
*Personal history of other health conditions*					
Thyroid disease	1	4	1	5	0.951
Lung disease	0	0	1	5	0.301
Asthma	5	20	1	5	0.106
Cancer	6	24	1	5	0.090
Kidney disease	1	4	1	5	0.951
					
*Signs & symptoms of CVD*					
Chest pain	13	52	10	45	0.565
Dyspnea	14	56	11	50	0.581
Heart palpitations	7	28	7	32	0.849
Skipped heart beats	8	32	8	36	0.834
Heart murmur	4	15	4	18	0.895
Intermittent leg-pain	10	40	11	50	0.581
Syncope	11	44	9	41	0.743
Fatigue	12	48	11	50	0.894
Snoring	12	48	12	53	0.764
Back pain	11	44	7	32	0.342
					
*Lifestyle factors*					
Current Smoker	0	0	1	5	0.301
Duration smoking (y)			54		
Previous Smoker	10	44	17	77	0.466
Quit duration (y)	21±16		26±15		
Alcohol consumption	12	48	15	74	0.220
Current weight loss plan	4	8	2	5	0.457
Everyday activity: sedentary	8	32	11	56	0.122
Light	14	56	10	45	
Moderate	3	12	1	5	
Vigorous	0	0	0	0	

Abbreviations: CVD, cardiovascular disease; TIA, transient ischaemic attack.

Note: heart problems refer to myocardial infarction, coronary artery disease and congestive heart failure. *P*-values are included to demonstrate no significant differences between groups at baseline.

**Table 2:** Number and percentage of people taking medication at baseline and PI for both conditions (exercise and control)

	*Exercise*		*Control*		*Baseline*	*PI*
	*Baseline*	*PI*	*Baseline*	*PI*	*Difference Ex* *vsCon (P)*	*Difference Ex* *vs* *Con (P)*
*Statins*						
*n*	21	18	17	17	0.329	0.830
%	84	72	77	77		
*Anti-thrombotic*^a^						
*n*	21	17	21	20	0.174	0.050^b^
%	84	68	95	91		
						
*Angiotensin converting enzyme inhibitor*						
* n*	13	13	12	13	0.968	0.724
%	52	52	55	59		
						
*Diuretics*						
*n*	2	1	2	2	0.926	0.508
%	8	4	9	9		
						
*Calcium blockers*						
*n*	2	2	5	5	0.179	0.179
%	8	8	23	23		
						
*Beta blockers*						
*n*	4	4	5	5	0.608	0.608
%	16	16	23	23		
						
*Anticoagulants*						
*n*	1	1	2	2	0.345	0.345
%	4	4	9	9		
						
*Angiotensin receptor blocker*						
*n*	2	1	0	0	0.174	0.160
%	8	4	0	0		
						
*Other anti-hypertensives*						
*n*	2	2	2	2	0.926	0.926
%	8	8	9	9		
						
*Mean medication use*						
X	3.78	3.08	3.76	3.76	0.947	0.094
s.d.	1.04	1.53	1.00	1.04		

Abbreviations: Con, control; Ex, exercise group; PI, post-intervention.

Mean (±s.d.) medication use is also reported.

a Number of patients taking two anti-thrombotics were *n*=18 (exercise, baseline), *n*=19 (control, baseline), *n*=8 (exercise, PI), *n*=14 (control, PI).

b Significant difference between groups (*P*<0.05).

**Table 3:** Mean (±s.d.) values for peripheral and central hemodynamic variables at baseline and PI for both conditions (exercise and control)

	*Exercise*		*Control*		*P*	*η*_*p*_^*2*^
	*Baseline*	*PI*	*Baseline*	*PI*		
*MAP (mm Hg)*						
X	96	90	98	96	0.160	0.05
s.d.	13	12	14	15		
						
*pDBP (mm Hg)*						
X	77	73	77	75	0.518	0.01
s.d.	10	10	9	11		
						
*pSBP (mm Hg)*						
X	134	125	140	138	0.046^a^	0.09
s.d.	21	19	27	28		
						
*cSBP (mm Hg)*						
X	127	118	133	132	0.027^a^	0.11
s.d.	19	17	26	28		
						
*pPP (mm Hg)*						
X	57	52	62	63	0.037^a^	0.10
s.d.	14	12	22	24		
						
*cPP (mm Hg)*						
X	49	43	53	55	0.011^a^	0.14
s.d.	13	10	21	23		
						
*pSBP-cSBP (mm Hg)*						
X	6.4	6.8	6.8	6.1	0.419	0.02
s.d.	4.9	3.7	4.8	4.4		
						
*AIx (%)*						
X	119	104	102	115	0.045^a^	0.09
s.d.	47	49	43	67		
						
*Heart rate (b.p.m.)*						
X	59	59	65	62	0.406	0.02
s.d.	9	12	16	14		
						
*DP*						
X	7591	7429	7795	8685	0.468	0.01
s.d.	2359	1985	3737	3111		

Abbreviations: AIx, augmentation index; b.p.m., beats per minute; cPP, central pulse pressure; cSBP, central systolic blood pressure; DP, double product; MAP, mean arterial blood pressure; pDP, peripheral double product; pDBP, peripheral diastolic blood pressure; PI, post-intervention; pPP, peripheral pulse pressure; pSBP, peripheral systolic blood pressure.

a Significant difference between groups (*P*<0.05).
