# Supplementary material for: A randomized controlled trial to assess the central hemodynamic response to exercise in patients with transient ischaemic attack and minor stroke
Source: J Hum Hypertens. 2016 Sep 29;31(3):172–7. doi: 10.1038/jhh.2016.72 (PMC5301081; doi:10.1038/jhh.2016.72)
Supplement: Supplementary Information [file jhh201672x1.docx]

**Supplementary Table** Mean (±SD) cardiovascular disease risk factors (excluding blood pressure) at baseline and post-intervention (PI) for both conditions (exercise, control)

|  |  | Exercise |  | Control |  | *P* | *η*_p_^2^ |
| --- | --- | --- | --- | --- | --- | --- | --- |
|  |  | Baseline | PI | Baseline | PI |  |  |
| TC (mmol/L) | X | 4.04 | 3.81 | 4.17 | 3.97 | 0.72 | 0.01 |
|  | SD | 1.07 | 1.05 | 0.97 | 1.00 |  |  |
| HDL (mmol/L) | X | 1.11 | 1.15 | 1.13 | 1.15 | 0.50 | 0.01 |
|  | SD | 0.30 | 0.32 | 0.30 | 0.32 |  |  |
| TC: HDL ratio | X | 3.73 | 3.45 | 3.89 | 3.73 | 0.59 | 0.01 |
|  | SD | 1.12 | 1.26 | 1.33 | 1.35 |  |  |
| FBG (mmol/L) | X | 5.58 | 5.58 | 6.45 | 6.57 | 0.54 | 0.01 |
|  | SD | 1.09 | 1.48 | 1.77 | 1.61 |  |  |
| Weight (kg) | X | 88 | 88 | 90 | 89 | 0.42 | 0.02 |
|  | SD | 19 | 19 | 21 | 21 |  |  |
| WC (cm) | X | 102 | 101 | 104 | 104 | 0.31 | 0.03 |
|  | SD | 15 | 15 | 19 | 18 |  |  |
| HC (cm) | X | 104 | 107 | 103 | 101 | 0.38 | 0.02 |
|  | SD | 17 | 16 | 19 | 22 |  |  |
| WC: HC ratio | X | 0.98 | 0.94 | 0.99 | 0.97 | 0.32 | 0.03 |
|  | SD | 0.09 | 0.10 | 0.09 | 0.10 |  |  |

*Abbreviations:* FBG, fasting blood glucose; HC, hip circumference; HDL, high-density lipoprotein;TC, total cholesterol; WC, waist circumference
